# Fixation eye movement abnormalities and stereopsis recovery following strabismus repair

**DOI:** 10.1038/s41598-021-93919-w

**Published:** 2021-07-13

**Authors:** Talora L. Martin, Jordan Murray, Kiran Garg, Charles Gallagher, Aasef G. Shaikh, Fatema F. Ghasia

**Affiliations:** 1 Department of Neurology, Case Western Reserve University, Cleveland, OH USA; 2 Visual Neurosciences and Ocular Motility Laboratory, Cole Eye Institute, Cleveland Clinic, Cleveland, OH USA; 3 Daroff-Del’Osso Ocular Motility Laboratory, Cleveland VA Medical Center, Cleveland, OH USA; 4 Neurology Service, Louis Stokes Cleveland VA Medical Center, Cleveland, OH USA

**Keywords:** Eye abnormalities, Vision disorders, Biomarkers

## Abstract

We evaluated the effects of strabismus repair on fixational eye movements (FEMs) and stereopsis recovery in patients with fusion maldevelopment nystagmus (FMN) and patients without nystagmus. Twenty-one patients with strabismus, twelve with FMN and nine without nystagmus, were tested before and after strabismus repair. Eye-movements were recorded during a gaze-holding task under monocular viewing conditions. Fast (fixational saccades and quick phases of nystagmus) and slow (inter-saccadic drifts and slow phases of nystagmus) FEMs and bivariate contour ellipse area (BCEA) were analyzed in the viewing and non-viewing eye. Strabismus repair improved the angle of strabismus in subjects with and without FMN, however patients without nystagmus were more likely to have improvement in stereoacuity. The fixational saccade amplitudes and intersaccadic drift velocities in both eyes decreased after strabismus repair in subjects without nystagmus. The slow phase velocities were higher in patients with FMN compared to inter-saccadic drifts in patients without nystagmus. There was no change in the BCEA after surgery in either group. In patients without nystagmus, the improvement of the binocular function (stereopsis), as well as decreased fixational saccade amplitude and intersaccadic drift velocity, could be due, at least partially, to central adaptive mechanisms rendered possible by surgical realignment of the eyes. The absence of improvement in patients with FMN post strabismus repair likely suggests the lack of such adaptive mechanisms in patients with early onset infantile strabismus. Assessment of fixation eye movement characteristics can be a useful tool to predict functional improvement post strabismus repair.

## Introduction

Normally, the two eyes see slightly different images (binocular disparity), however the stimulation of the respective retina of each eye produces a common subjective visual perception of fusion. The achievement of fusion is complex as our eyes are never completely still due to the occurrence of physiologic involuntary fixational eye movements (FEMs) namely the fixational saccades, inter-saccadic drift, and ocular tremor. The FEMs between the two eyes are conjugate with similar amplitude and direction^1–3^. Thus, despite the constant motion of the eyes, normal FEMs do not prevent binocular fusion, suggesting close coordination between the visual sensory and motor systems. Strabismus interferes with visual-motor coordination of eye movements, resulting in disconjugate and cross-axis eye movements during visually guided saccades, variable and subnormal vergence responses, and fixation instability^4–7^.

Non-human primate (NHP) studies have shown that disruption of binocularity during infancy is often associated with development of fusion maldevelopment nystagmus syndrome (FMNS) and loss of stereopsis^8–10^. These studies have revealed that loss of binocular connections within V1 in the first months of life is the necessary and sufficient cause of FMN, and that the prevalence and severity of FMNS increases with the longer duration of binocular de-correlation^11^. NHP studies have also provided important insights into the neural correlates within the brainstem that result in the oculomotor abnormalities associated with strabismus^12–15^. Following strabismus repair in NHPs, there were significant changes in the magnitude of visually guided saccades with small changes in fixational stability of the viewing and non-viewing eye.

Studies in patients have shown that the disconjugacy of visually guided saccades decreases, with improvement of vergence responses after surgical strabismus repair^16,17^. We have previously shown that the fixation instability arises due to presence of nystagmus and abnormalities of fixational saccades with increased inter-saccadic drifts^7,18,19^. We have found an increase in the amplitude of fixational saccades of the viewing and non–viewing eye and an increase in the variance of eye position of the non-viewing eye, which was worse in patients with large-angle strabismus and poor stereopsis. In this paper, we will examine the impact of strabismus repair on FEM abnormalities of the viewing and non-viewing eye, and whether the presence of FMN impacts surgical outcomes. We hypothesize that (a) the presence of nystagmus will be associated with poor stereopsis recovery post strabismus repair despite improvement in eye alignment and (b) patients without nystagmus are more likely to have improvement of fast and slow FEM abnormalities of the viewing and non-viewing eye due to adaptive mechanisms promoted by realignment of the eyes and recovery of stereopsis.

## Methods

The Cleveland Clinic Institutional Review Board approved the protocol and written informed consent was obtained from each participant or parent/legal guardian in accordance with the Declaration of Helsinki. We performed a review of subjects who were recruited for eye movement recordings in the lab and identified twenty-one subjects who had measurements from before and at least 2 months after strabismus repair (Table 1). The clinical parameters were extracted from a chart review for all the enrolled subjects. None of the subjects had any structural anomalies of the eye or neurologic disorders or cranial nerve palsies. Patients had received treatment according to the American Academy of Ophthalmology Preferred Practice Pattern. The type and age of prior strabismus repair was noted. The visual acuity and stereo-acuity, cycloplegic refraction, strabismus angle measurements at distance and near at the time of eye movement recordings were noted. Visual acuity was measured in each eye monocularly, starting from the right eye, using the participant’s optimal spectacle correction with Snellen linear optotype. Visual acuity was measured at 20 feet distance, and the value was considered only if the patient could read all the letters (or symbols) of the line. Stereo-acuity was measured with the Titmus Stereo Test at 40 cm. For the purpose of analysis, subjects with no detectable (nil) stereo-acuity were assigned a value of 7000″. The visual acuity scores were converted into logMAR values, and stereo-acuity scores in seconds of arc were converted to log arcsec values for statistical analysis.

**Table 1:** The clinical and demographic features of subjects included in the study.

ID	Gender	Nystagmus	Acuity OD (LogMAR)	Acuity OS (LogMAR)	Preop stereoacuity (logarc sec)	Postop stereoacuity (logarc sec)	Refraction OD	Refraction OS	Strabismus near	Strabismus distance	Age at Current Surgery	Current surgery	Prior surgeries
1	F	None	0.00	0.00	3.85	3.85	− 5.25	− 2.75 + 1.0 × 170	16 XT, 6 LHT	14 XT	46	LMR advance, LIO myectomy	Yes × 1 (age 3 BMR recess)
2	F	None	0.00	0.00	3.85	1.78	+ 3.0	+ 3.0	45 ET	45 ET	7	BMR recess	None
3	F	None	0.00	0.00	2.30	1.70	− 1.75 + 1.75 × 90	+ 0.25 + 0.5 × 70	25 ET	25 E(T)	58	BLR advance, transposition	Yes (age 4 BLR recess)
4	F	None	0.00	0.00	3.85	2.60	− 1.25 + 1.0 × 111	+ 0.25 + 0.50 × 86	25 X(T)	25 X(T)	15	BLR recess	Yes × 2 (age 8 LMR recess; age 13 LMR advance)
5	F	None	− 0.12	− 0.12	1.90	1.78	− 5.5	− 6.0	25 ET	30 E(T)	31	BMR recess	None
6	M	None	0.00	0.00	2.00	1.60	− 7.25	+ 7.5 + 0.5 × 168	35 ET, 1 LHT	25 ET	18	BMR recess	None
7	F	None	0.00	0.00	1.70	1.60	Plano	Plano	20 XT	30 X(T)	7	BLR recess, Bilat IO myectomy	None
8	F	None	− 0.12	0.00	3.54	2.15	− 6.75 + 0.5 × 94	− 6.5 + 1.25 × 40	30 ET	16ET	20	BMR recess	None
9	M	None	0.00	0.00	3.85	1.60	− 7.0 + 2.00 × 103	− 6.75 + 0.75 × 66	35 ET	35 ET	52	BMR recess	None
10	M	Nystagmus	0.40	0.10	3.85	3.85	+ 3.5 + 1.5 × 110	+ 1.0 + 0.5 × 80	50 ET	50 ET	14	RMR recess, RLR resect	None
11	M	Nystagmus	0.00	0.00	2.00	1.78	− 2.5 + 1.5 × 105	− 1.75 + 0.75 × 85	30 X(T), 5 RHT	25 X(T)	13	BLR recess	None
12	M	Nystagmus	0.00	0.18	3.85	3.54	+ 3.5	+ 4.5	2 ET, 15 LHT, L DVD	12 ET	11	LIO anteriorization	Yes × 1 (age 1.5 BMR recess)
13	F	Nystagmus	0.18	0.00	3.85	3.85	Plano	− 0.50 + 0.25 × 25	35 XT	35 XT	43	RLR recess, RMR resect, RIO myectomy	Yes × 1 (age 2 years BMR recess)
14	F	Nystagmus	0.18	0.10	3.54	2.90	+ 4.0	+ 2.25	30 X(T), 5 RHT	25 X(T), small LHT	6	BLR recess, bilat IO myectomy	None
15	F	Nystagmus	0.10	0.30	3.85	3.85	+ 0.25 + 0.5 × 100	− 1.25 + 4.75 × 40	40 XT	40 XT	42	RLR recess, RMR resect	Yes × 2 (age 2.5 years: LMR recess, age 41: LLR recess, LMR advance)
16	M	Nystagmus	0.10	0.40	3.85	3.85	− 6.5 + 3.5 × 80	− 11.5 + 4.75 × 85	16 XT, 8 RHT	16 XT, 8 RHT	9	BLR rerecess, BIO myectomy	Yes × 1 (age 7: BLR recess)
17	F	Nystagmus	0.10	0.30	3.85	3.85	+ 0.25	+ 0.25	45 XT, bil DVD	35 X(T)	39	BMR advance	Yes × 1 (age 2 BMR recess)
18	M	Nystagmus	− 0.12	− 0.12	3.85	3.85	− 0.25 + 0.75 × 13	+ 0.50 + 1.0 × 172	30 XT	35 XT	43	BLR recess	None
19	F	Nystagmus	0.10	0.10	3.85	3.85	Plano	Plano	20 ET, 4 LHT	20 ET, 4 LHT	69	RLR resect + advance	Yes × 2 (age 2 BMR recess and age 49 BLR recess)
20	F	Nystagmus	0.48	0.00	3.85	3.85	+ 3.5 + 0.5 × 30	+ 1.75	30 ET, 8 RHT	30 ET, 8 RHT	37	RMR recess, and RLR resect with RIO myectomy	None
21	F	Nystagmus	0.00	0.18	3.85	3.85	− 0.25	+ 3.5 + 0.5 × 96	30 XT, bil DVD	30 XT, bil DVD	15	BLR recess, Bilat IO myectomy	None

*F* female, *M* male, *ET* esotropia, *XT* exotropia, *DVD* dissociated vertical deviation. () = intermittent deviation.

*HT* hypertropia, *LR* lateral recti muscles, *MR* medial recti muscles, *IO* inferior oblique. (Preceded by B- Bilateral, L- Left, R- Right).

We report the strabismus angle, stereoacuity, monocular visual acuity measured at the time of eye movement recordings before and after strabismus repair.

### Eye movement recordings

We have previously used these methodology and data analysis techniques in our prior publications^7,19,20^. High-resolution video-based eye tracker (EyeLink 1000, SR Research, Ontario, Canada) was used to non-invasively measure horizontal and vertical eye position in subjects with strabismus. Subject’s head was supported on a chin rest, 55 cm away from the liquid–crystal display screen. The subjects were instructed to fixate their gaze on a circular visual target (the diameter subtended 0.5° visual angle) projected on the screen with a white background in a completely dark room for 45 s. We recorded binocular eye positions under monocular viewing conditions by using an infrared filter, which blocks the visible light over one eye. Each eye was calibrated before the beginning of a trial. Subjects wore the corrective lenses for the experiments.

### Data analysis

The trials were categorized as fellow eye viewing and amblyopic eye viewing conditions. In patients without amblyopia, we determined if subjects had a fixation preference and designated the fixing eye as fellow eye and analyzed the non-fixing eye as the amblyopic eye. Eye position data were used for further analysis. Blinks and partial blinks were identified and removed^21,22^. The eye position signal was differentiated using Matlab (Mathworks, Natick, MA) differential function and was further smoothened with the Savitzkey-Golay filter to measure eye velocity.

We separately analyzed subjects without nystagmus and subjects with nystagmus, with methods previously used in our publications^7,19,20,23^. Fixational saccades in patients without nystagmus were defined as saccades produced during attempted fixation and quick phases in patients with nystagmus were identified using the unsupervised clustering method. Fixational saccades/quick phase amplitude was defined as the absolute difference between the eye positions at the start and at the end of the fast eye movement. Small rapid eye movement in the opposite direction called dynamic overshoot follows some saccades/quick phases. We identified the dynamic overshoot by their very short latency (< 20 ms) between the two movements and were not considered as a “new” saccade. In some occasions, the quick phases were followed by dynamic overshoot followed by ringing which were more pronounced in the non-viewing eye. Such movements when present were removed from analysis. Drifts in patients without nystagmus and slow phases in patients with nystagmus were defined as epochs between fixational saccades and quick phases respectively. We removed 20 ms from the start and end of each of these epochs to exclude periods of acceleration and deceleration of the eye during fixational saccades/quick phases and blinks. The amplitude during fixational saccades/quick phases, median eye velocity during inter-saccadic drifts/slow phases was computed for the viewing eye (VE) and non-viewing eye (NVE). To determine whether strabismus repair has an effect on fast and slow fixation eye movements, we computed the percentile (10th, 25th, 50th, 75th and 90th) of the amplitude of fast eye movements of the viewing eye and non-viewing eye for each subject during fellow eye viewing and amblyopic eye viewing condition. A similar analysis was done on the median eye velocities of the slow fixational eye movements. Fixational saccades and quick phases frequency was computed as a number of events in one second.

We also quantified the fixation stability by measuring a bivariate contour ellipse (BCEA) using the following equation^7,24^.

$$ {\text{BCEA}} = \pi {\text{ }}{\rm X}^{2} \sigma _{x} \sigma _{y} \sqrt {1 - p2} $$

In the equation Χ^2^ is a chi-square variable with two degrees of freedom, σ _x_ σ_y_ are the standard deviation of eye position in the horizontal and vertical meridian respectively, and *p* is the product moment correlation of the two position components. The area of the 95% bivariate contour ellipse (i.e. BCEA, in deg^2^) was used in this study as a quantitative measure of fixation instability. A log_10_ transformation was used to normalize the resulting BCEA values. All the parameters were measured for the VE and NVE. All the data analysis was performed using custom prepared software in Matlab programming language.

### Statistical analysis

The statistical analysis was performed using GraphPad Prism six and SPSS software. Paired t-tests were used to compare the change in strabismus angle after strabismus repair in all subjects. Fisher exact tests were used to compare total number of surgeries and presence of amblyopia in subjects with and without nystagmus. An unpaired t-test was performed to compare age at surgery (before and after which eye movement recordings were obtained), and to determine the extent of improvement in strabismus angle and stereopsis recovery after strabismus repair in patients with and without nystagmus. A two-way mixed ANOVA (one within and one between subjects factor) was run to determine the effect of strabismus repair on visual acuity and fixation instability as measured by log BCEA in patients without and with nystagmus. Mauchly’s test of sphericity was conducted to test the null hypothesis that the variances of differences between all combinations of related groups (levels) of the ANOVA test are equal. If Mauchly's Test of Sphericity is statistically significant (*p* < 0.05), we would reject the null hypothesis and accept the alternative hypothesis that the variances of the differences are not equal (i.e., sphericity has been violated). For the ANOVA tests, Mauchly's test of sphericity had p value > 0.05 indicating that the assumption of sphericity was met. A Wilcoxon matched pair test was used to compare the frequency of quick phases and fixational saccades before and after strabismus repair. A Wilcoxon matched pair test was also used to compare the percentile data of the amplitude of fast eye movements of the viewing/fixing eye and non-viewing/non-fixing eye before and after strabismus repair. A similar analysis was done on the percentile data of the median horizontal velocities of slow eye movements before and after strabismus repair. For all statistical tests, significance was defined as p < 0.05.

## Results

**Figure 1:**
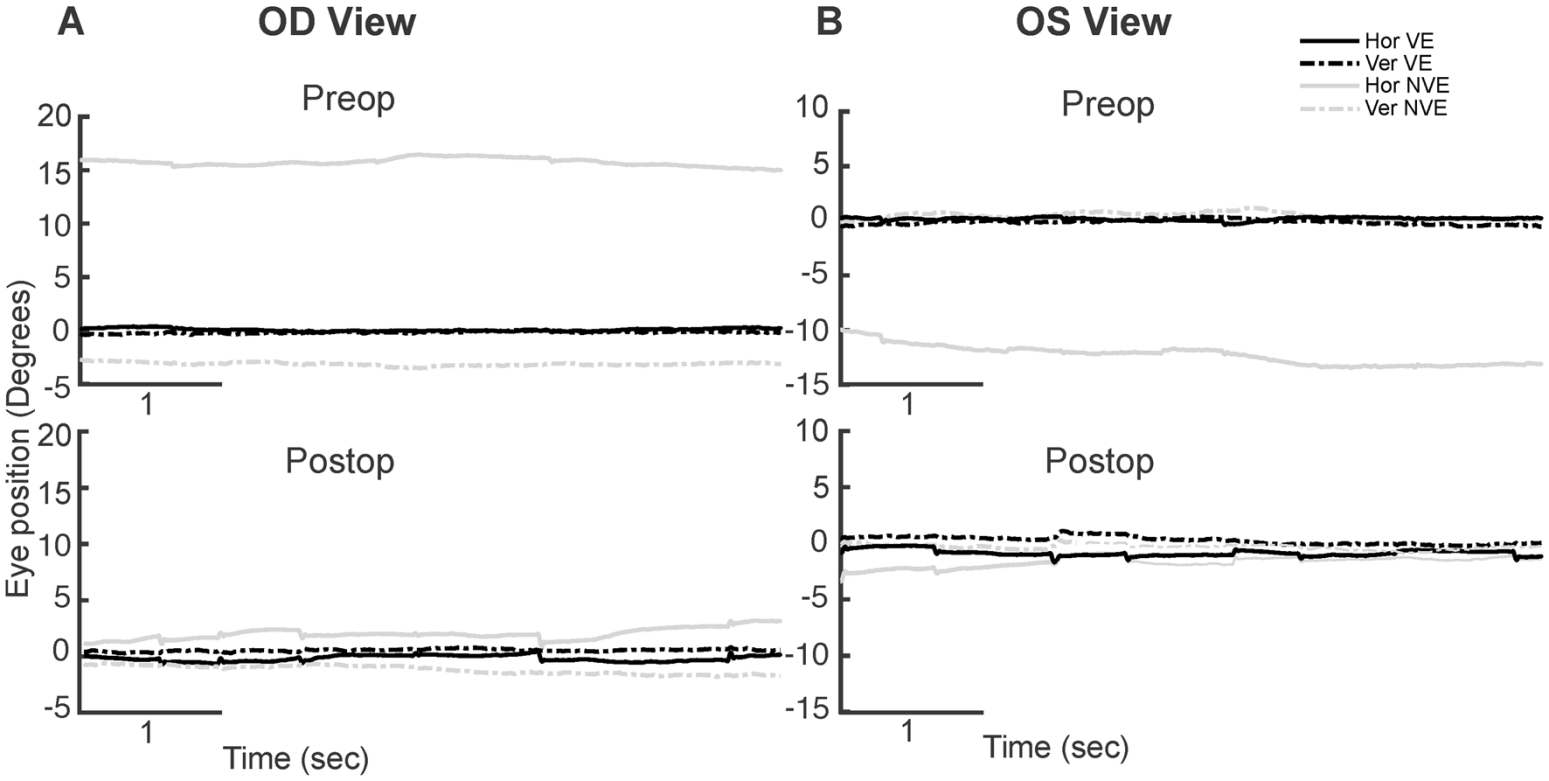
Examples of visual fixation during a 5 s epoch under conditions of monocular viewing from a subject with no nystagmus, pre- and post- strabismus repair during right eye viewing (OD viewing—**A**) and left eye viewing (OS viewing—**B**), pre- and post- strabismus repair. The x-axis represents time and the y-axis represents horizontal (solid line, black: viewing eye, grey: non-viewing eye) and vertical (dotted line, black: viewing eye, grey: non-viewing eye) positions.

**Figure 2:**
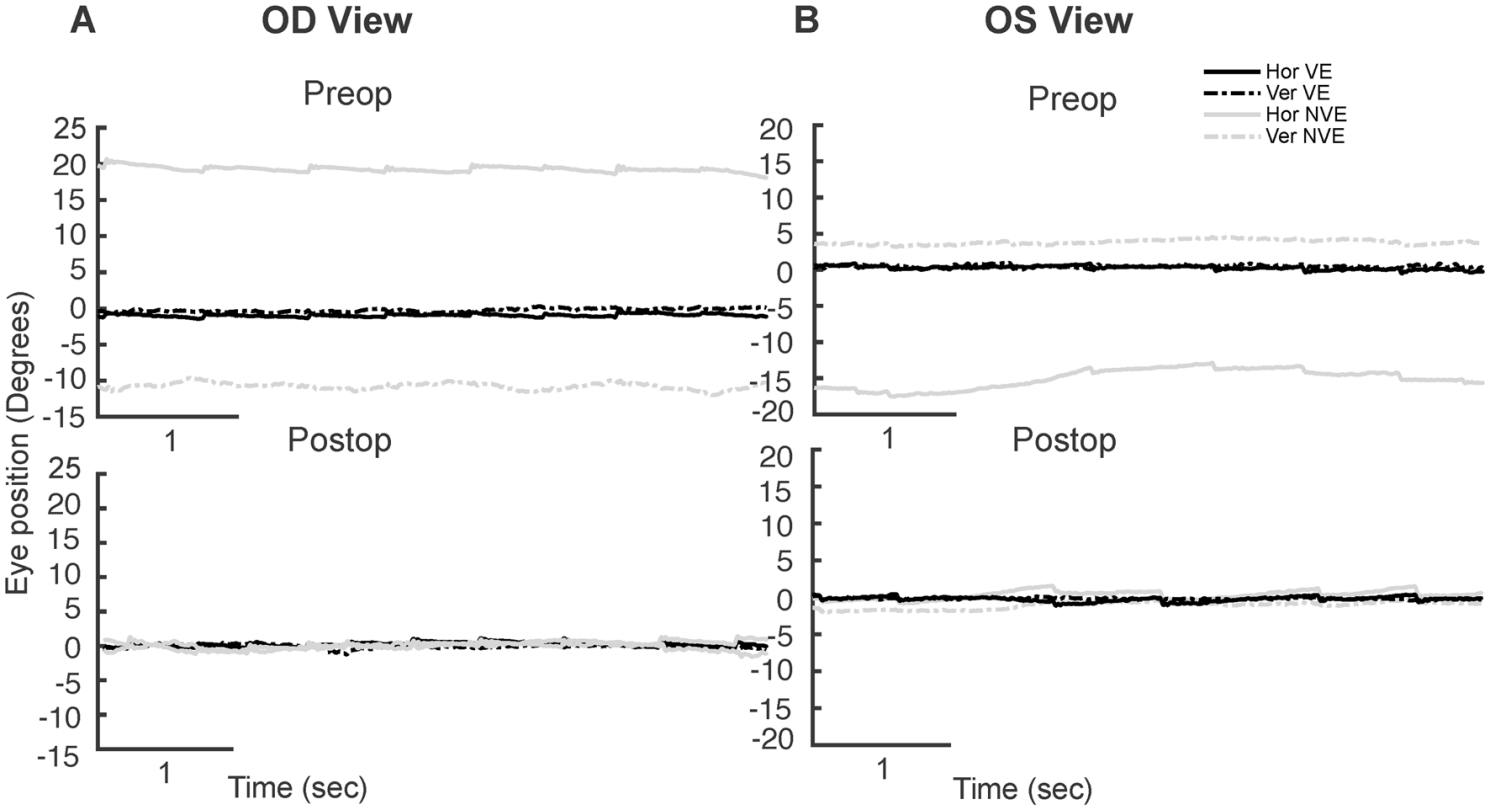
Examples of visual fixation during a 5 s epoch under conditions of monocular viewing from a subject with fusion maldevelopment nystagmus, pre- and post- strabismus repair during right eye viewing (OD viewing—**A**) and left eye viewing (OS viewing—**B**) pre- and post- strabismus repair. The x-axis represents time and the y-axis represents horizontal (solid line, black: viewing eye, grey: non-viewing eye) and vertical (dotted line, black: viewing eye, grey: non-viewing eye) positions.

Patients with strabismus have increased fixation disparity with misalignment between the right and left eyes and abnormal binocular function. They also have fixation instability that arises due to the presence of nystagmus versus alterations in the physiologic involuntary fixation eye movements^7^. We evaluated the FEM traces and classified the patients based on the presence or absence of nystagmus (Fig. 1). Figure 1A,B plots the FEMs obtained during a 5 s epoch in a patient without nystagmus before and after strabismus repair (subject 5). Patients without nystagmus exhibited alternating fixational saccades (black arrows) with inter-saccadic drifts (brackets), similar to healthy subjects^18,25,26^. Figure 2A,B plots a 5 s epoch of FEMs in a patient with nystagmus (subject 19) before and after strabismus repair. The presence of FMNS was determined based on the classic reversal in the direction of the quick phase of nystagmus (black arrows) with linear/decreasing velocity nasally directed slow phases (brackets) observed during monocular viewing conditions. The quick phases were directed temporally whereas the slow phases were directed nasally. Notice the reduction in angle of strabismus before and after strabismus repair in both patients.

**Figure 3:**
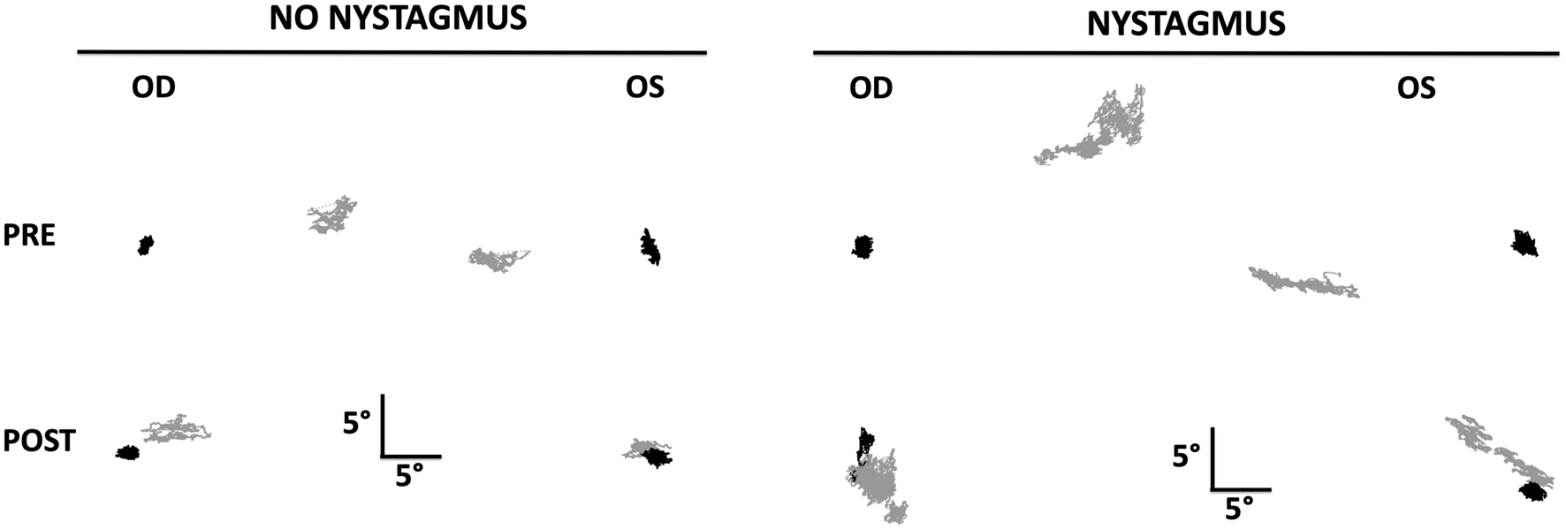
Representative fixation plots pre- and post- strabismus repair, from a subject with no nystagmus and a subject with nystagmus. Gray = non-viewing eye, Black = viewing eye.

Figure 3 plots the scatter plot of horizontal and vertical eye positions in a patient with and without nystagmus during right eye viewing and left eye viewing condition indicating the eye misalignment before and after strabismus repair. Both patients had esotropia preoperatively—the scatter plot of eye positions of the left eye are shifted to the right during right eye viewing and the scatter plot of eye positions of the right eye are shifted to the left during left eye viewing. In addition, the patient with nystagmus had a vertical eye misalignment during right and left eye viewing preoperatively, which was most evident during right eye viewing condition (suggestive of dissociated vertical deviation with superimposed small left hypertropia). There was a significant improvement in the eye misalignment postoperatively in both subjects with marked improvement in esotropia as well as the vertical misalignment in the patient with nystagmus. We quantified the fixation stability by calculating the bivariate contour ellipse (BCEA) of the VE and NVE. The log10 [BCEA (deg^2^)] of the NVE was greater both before and after strabismus repair in the patient without nystagmus [Preop OD viewing—OD: 0.03, OS: 0.96 and OS viewing—OS: 0.46, OD: 0.72; and Postop OD viewing—OD: 0.10, OS: 1.0 and OS viewing—OS: 0.24, OD: 0.46] and in the patient with nystagmus [Preop OD viewing—OD: 0.19, OS: 1.5 and OS viewing—OS: 0.99, OD: 0.23; and Postop OD viewing—OD: 0.69, OS: 1.19 and OS viewing—OS: 0.13, OD: 1.29]. We further quantified these results in the subsequent sections.

### Preoperative characteristics of FEMS and postoperative anatomical and functional outcomes

Table 1 lists the demographic features, and clinical characteristics of subjects at the time of recruitment in the study. We characterized the patients based on FEM characteristics into those without nystagmus (n = 9) and those with nystagmus (n = 12). Nine subjects required multiple surgeries (no nystagmus = 3, nystagmus = 6, fisher exact test p = 0.37). There was no difference in age at surgery, before and after which eye movement recordings were obtained, in patients without (28.2 ± 19.4) and with nystagmus (28.4 ± 19.7), unpaired t test (p = 0.982).

**Figure 4:**
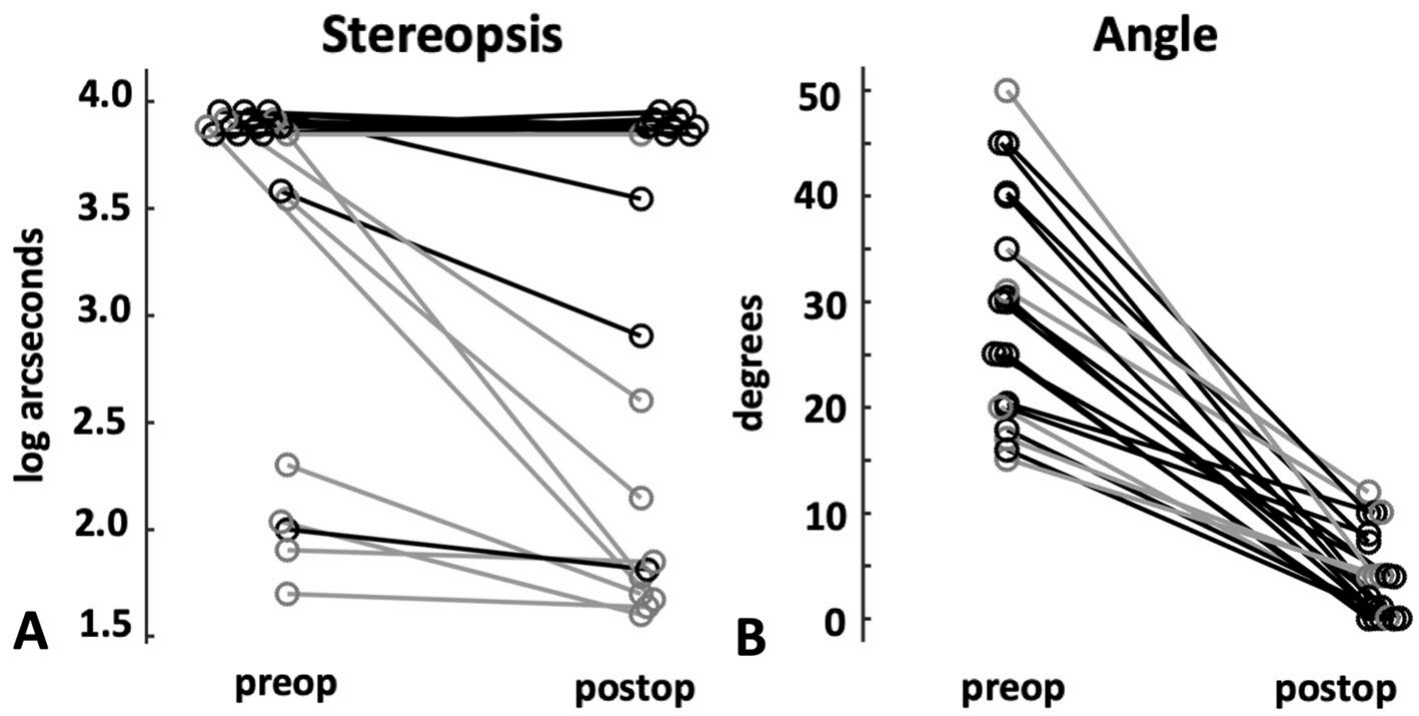
Pre-and postoperative strabismus angle (**A**) and stereopsis (**B**) in subjects with nystagmus (black) and in subjects without nystagmus (gray).

Figure 4A,B plots the composite angle of strabismus and stereopsis before and after strabismus repair. There was significant improvement with reduction in the strabismus angle (horizontal angle ≤ 10 PD and vertical angle ≤ 5 PD) after strabismus repair in patients without and with nystagmus (paired t test, p < 0.00001). The amount of improvement of strabismus angle i.e. the angle before and after strabismus repair was similar in patients with and without nystagmus (unpaired t test, p = 0.58). Figure 4B plots the stereopsis before and after strabismus repair. The stereopsis recovery i.e. difference in stereopsis before and after strabismus repair was greater in patients without nystagmus (0.9 ± 0.86 log arcsecs) with negligible recovery of stereopsis in patients in nystagmus (0.09 ± 0.2 log arcsecs) (unpaired t test, p = 0005).

None of the patients without nystagmus and 8 patients with nystagmus had amblyopia (n = 8, fisher exact test p = 0.002). A two-way mixed ANOVA (two independent variables: one between subject factor i.e., patients without and with nystagmus and one within subject factor i.e., before and after strabismus repair) was run to determine the effect on visual acuity (dependent variable) of fellow eye. Mauchly's test of sphericity indicated that the assumption of sphericity was met. There was no statistically significant main effect on visual acuity of the fellow eye before and after strabismus repair *F* (1, 19) = 0.98, *p* = 0.33, partial η^2^ = 0.049. There was also no interaction between the strabismus repair and fellow eye visual acuity in patients without and with nystagmus, *F* (1, 19) = 0.9, *p* = 0.3, partial η^2^ = 0.05. The visual acuity of the fellow(fixing) eye before strabismus repair was better in patients without nystagmus compared to those with nystagmus (w/o nystagmus: -0.02 ± 0.05 logMAR, w nystagmus: 0.03 ± 0.07 logMAR, F (1,19) = 5.3, p = 0.03, partial η^2^ = 0.22). The visual acuity of the fellow (fixing) eye after strabismus repair was better in patients without nystagmus compared to those with nystagmus (w/o nystagmus: -0.02 ± 0.05 logMAR, w nystagmus: 0.02 ± 0.07 logMAR, F (1,19) = 2.6, p = 0.18, partial η^2^ = 0.11).

A two-way mixed ANOVA (two independent variables: one between subject factor i.e., patients without and with nystagmus and one within subject factor i.e., before and after strabismus repair) was run to determine the effect on visual acuity (dependent variable) of amblyopic (non-fixing) eye. Mauchly's test of sphericity indicated that the assumption of sphericity was met. There was no statistically significant main effect on visual acuity of the amblyopic (non-fixing) eye before and after strabismus repair *F* (1, 19) = 3.34, *p* = 0.08, partial η^2^ = 0.15. There was also no statistically significant interaction between the strabismus repair and amblyopic eye visual acuity in patients without and with nystagmus, *F* (1, 19) = 3.32, *p* = 0.08, partial η^2^ = 0.15. The visual acuity of the amblyopic (non-fixing) eye before strabismus repair was better in patients without nystagmus compared to those without nystagmus (w/o nystagmus: -0.01 ± 0.04 logMAR, w nystagmus: 0.19 ± 0.18 logMAR, F (1,19) = 11.3, p = 0.003, partial η^2^ = 0.37). The visual acuity of the amblyopic (non-fixing) eye after strabismus repair was better in patients without nystagmus compared to those without nystagmus (w/o nystagmus: -0.01 ± 0.04 logMAR, w nystagmus: 0.15 ± 0.16 logMAR, F (1,19) = 8.6, p = 0.008, partial η^2^ = 0.31).

### Fast eye movements of the VE and NVE before and after strabismus repair

#### Frequency of fast eye movements

A Wilcoxon matched pair test was run to determine the effect of strabismus repair on frequency of fixational saccades and quick phases in patients without and with nystagmus respectively during fellow eye viewing condition. There was no statistically significant difference in the frequencies of fast eye movements in patients without nystagmus [Preop: 1.0 ± 0.49, Postop: 1.0 ± 0.3, p > 0.99] and in patients with nystagmus [Preop: 1.3 ± 0.42, Postop: 1.4 ± 0.6, p > 0.84]. A similar analysis was done during amblyopic eye viewing condition and no statistically significant difference was noted on the frequency of fixational saccades and quick phases in patients without nystagmus [Preop: 0.9 ± 0.39, Postop: 1.1 ± 0.2, p = 0.06] and with nystagmus [Preop: 1.9 ± 0.56, Postop: 1.7 ± 0.75, p = 0.46] respectively.

#### Amplitude of fast eye movements

**Figure 5:**
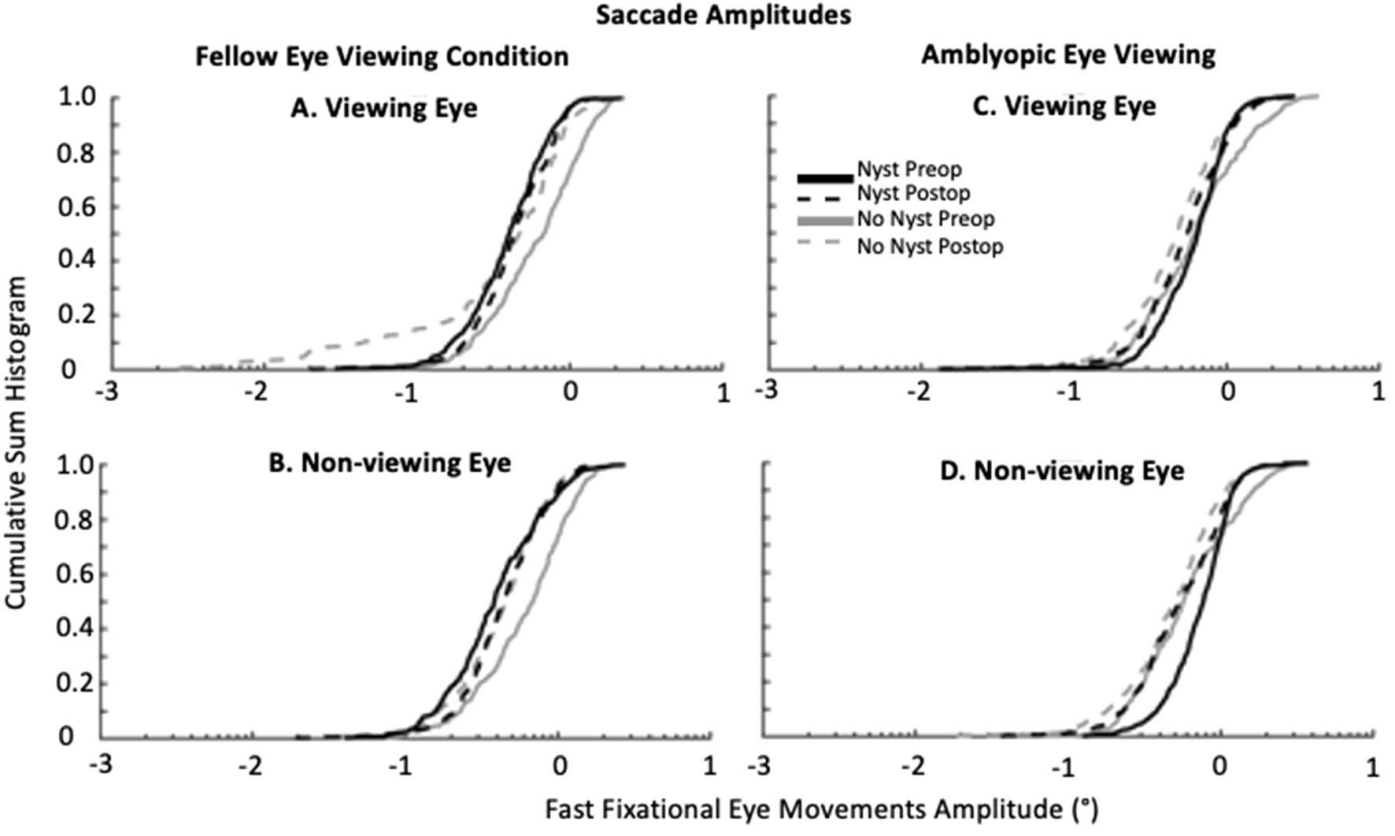
Cumulative sum histograms of fixational saccade amplitudes (°) of the viewing and non-viewing eye during fellow eye (**A** viewing eye, **B** non-viewing eye) and amblyopic eye (**C** viewing eye, **D** non-viewing eye) viewing conditions, produced during a gaze-holding task. Solid lines represent before (grey = no nystagmus, black = nystagmus), and dashed lines represent after strabismus repair (grey = no nystagmus, black = nystagmus). X-axis is log scale.

Figure 5 summarizes the normalized cumulative sum histogram of the fixational saccades and quick phases of the VE and NVE in patients without and with nystagmus respectively during fellow eye viewing (Fig. 5A,B) and amblyopic eye viewing conditions (Fig. 5C,D). There is a leftward shift of the distribution of amplitude of the fixational saccades of VE and NVE particularly during FEV in patients without nystagmus after strabismus repair. We computed the percentile of the amplitude of the VE and NVE for each subject. We then pooled these values and performed pairwise comparisons for before and after strabismus repair in patients without nystagmus and with nystagmus. We found that the amplitude of the VE and NVE were greater during FEV and AEV in patients without nystagmus prior to strabismus repair (Table 2). The leftward shift after strabismus repair was statistically significant for the VE for the 10th and 25th percentile and the 75th and 90th percentile for the NVE during FEV (fixing eye viewing) condition and 10th percentile for the VE during AEV (non-fixing eye viewing) condition (Table 2).

**Table 2:** Percentile amplitude (°) of fixational saccades in viewing eye and non-viewing eye (non-viewing eye in parenthesis) of subjects without and with nystagmus, before and after strabismus repair.

	Amblyopic eye viewing			Fellow eye viewing		
	Preop	Postop	p value	Preop	Postop	p value
**Amplitude (°) of fixational saccade—subjects without nystagmus**						
10th	0.34 ± 0.29 (0.34 ± 0.28)	0.21 ± 0.11 (0.25 ± 0.18)	0.039 (0.13)	0.30 ± 0.22 (0.31 ± 0.22)	0.19 ± 0.13 (0.21 ± 0.096)	0.039 (0.039)
25th	0.47 ± 0.33 (0.48 ± 0.35)	0.32 ± 0.12 (0.36 ± 0.19)	0.055 (0.16)	0.41 ± 0.27 (0.39 ± 0.24)	0.29 ± 0.21 (0.32 ± 0.13)	0.039 (0.23)
50th	0.67 ± 0.46 (0.65 ± 0.40)	0.49 ± 0.15 (0.50 ± 0.23)	0.16 (0.16)	0.56 ± 0.33 (0.58 ± 0.28)	0.47 ± 0.28 (0.47 ± 0.16)	0.19 (0.15)
75th	0.95 ± 0.62 (0.91 ± 0.56)	0.73 ± 0.20 (0.72 ± 0.24)	0.23 (0.19)	0.81 ± 0.39 (0.84 ± 0.36)	0.70 ± 0.25 (0.64 ± 0.16)	0.15 (0.039)
90th	1.25 ± 0.85 (1.22 ± 0.76)	1.07 ± 0.36 (1.11 ± 0.41)	0.47 (0.50)	1.02 ± 0.52 (1.13 ± 0.43)	0.89 ± 0.26 (0.86 ± 0.21)	0.19 (0.039)
**Amplitude (°) of quick phase—Nystagmus subjects**						
10th	0.29 ± 0.14 (0.40 ± 0.17)	0.29 ± 0.15 (0.34 ± 0.22)	0.50 (0.19)	0.22 ± 0.096 (0.24 ± 0.15)	0.26 ± 0.14 (0.26 ± 0.16)	0.16 (0.50)
25th	0.44 ± 0.18 (0.53 ± 0.21)	0.43 ± 0.14 (0.47 ± 0.23)	0.34 (0.23)	0.33 ± 0.14 (0.34 ± 0.23)	0.35 ± 0.16 (0.36 ± 0.19)	0.22 (0.31)
50th	0.56 ± 0.21 (0.68 ± 0.24)	0.58 ± 0.21 (0.64 ± 0.29)	0.23 (0.34)	0.44 ± 0.18 (0.49 ± 0.31)	0.47 ± 0.21 (0.47 ± 0.24)	0.38 (0.31)
75th	0.70 ± 0.23 (0.88 ± 0.23)	0.78 ± 0.26 (0.82 ± 0.31)	0.15 (0.41)	0.56 ± 0.19 (0.63 ± 0.34)	0.57 ± 0.23 (0.60 ± 0.30)	0.50 (0.28)
90th	0.85 ± 0.33 (1.12 ± 0.29)	1.04 ± 0.31 (1.11 ± 0.33)	0.055 (0.47)	0.71 ± 0.23 (0.82 ± 0.40)	0.71 ± 0.26 (0.76 ± 0.38)	0.50 (0.22)

### Slow fixation eye movements of the VE and NVE before and after strabismus repair

**Figure 6:**
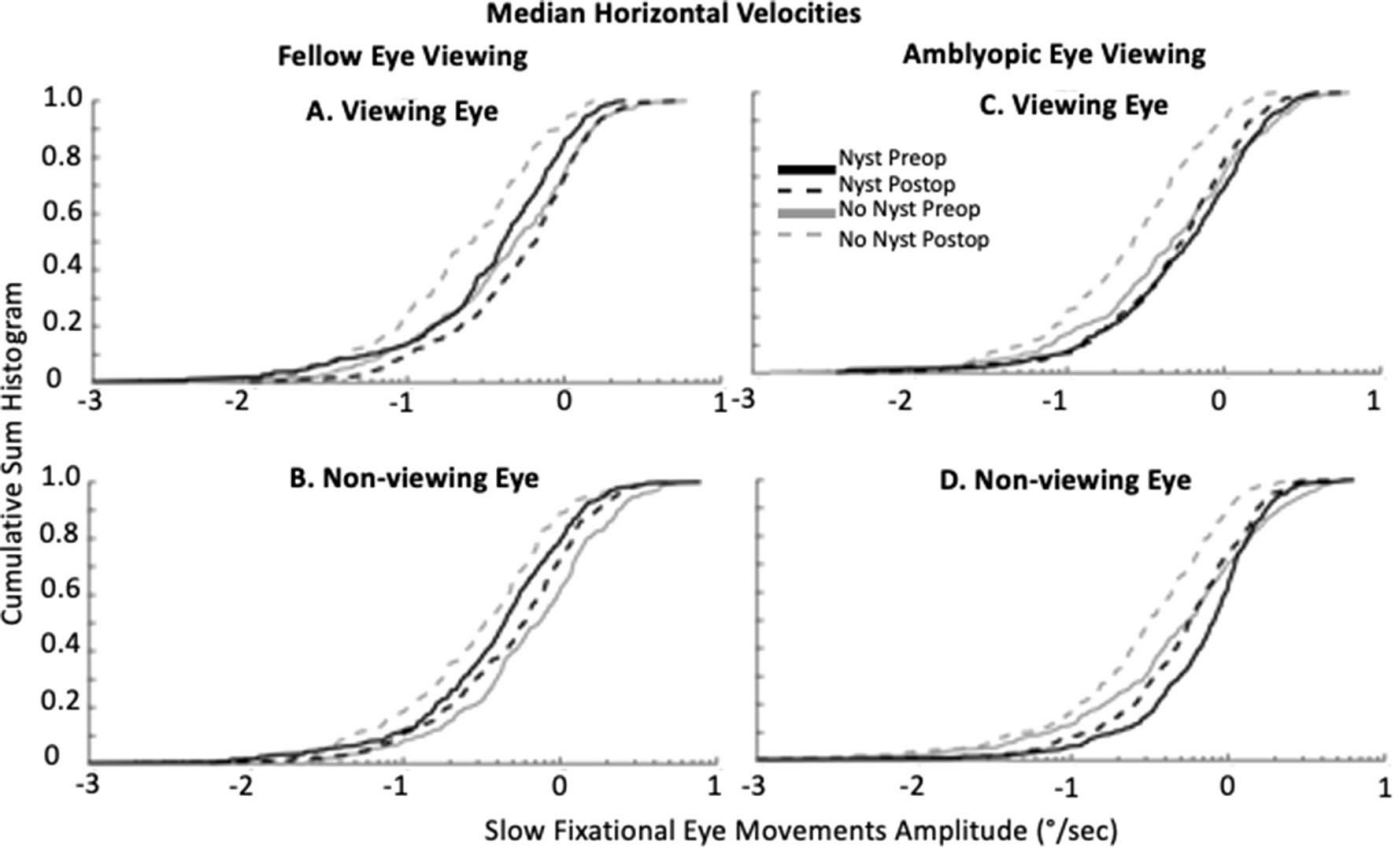
Cumulative sum histograms of median horizontal velocities (°/s) of the viewing and non-viewing eye during fellow eye (**A** viewing eye, **B** non-viewing eye) and amblyopic eye (**C** viewing eye, **D** non-viewing eye) viewing conditions, produced during a gaze-holding task. Solid lines represent pre-operative data (grey = no nystagmus, black = with nystagmus), and dashed lines represent post-operative data (grey = no nystagmus, black = with nystagmus). X-axis is log scale.

Figure 6 summarizes the normalized cumulative sum histogram of the median horizontal velocities of the inter-saccadic drift and slow phases of the VE and NVE in patients without and with nystagmus respectively during fellow eye viewing (Fig. 6A,B) and amblyopic eye viewing conditions (Fig. 6C,D). There is a leftward shift of the distribution of the median horizontal velocities of VE and NVE particularly during FEV and AEV in patients without nystagmus post strabismus repair. We computed the percentile of the median horizontal velocity of the VE and NVE for each subject. We then pooled these values and performed pairwise comparisons for before and after strabismus repair in patients without nystagmus and with nystagmus. We found that overall the velocities of the VE and NVE were greater during FEV and AEV in patients without nystagmus prior to strabismus repair (Table 3). The leftward shift after strabismus repair was statistically significant for the 90th percentile for the VE during FEV, 10th percentile for the VE during AEV and for the 25th and 50th percentile of the NVE during AEV condition (Table 3).

**Table 3:** Percentile horizontal drift velocity (°/s) in viewing eye and non-viewing eye (non-viewing eye in parenthesis) of subjects without and with nystagmus, before and after strabismus repair.

	Amblyopic eye viewing			Fellow eye viewing		
	Preop	Postop	p value	Preop	Postop	p value
**Horizontal drift velocity (°/s)—subjects without nystagmus**						
10th	0.075 ± 0.054 (0.057 ± 0.048)	0.047 ± 0.036 (0.057 ± 0.55)	0.0078 (0.42)	0.075 ± 0.043 (0.084 ± 0.082)	0.058 ± 0.045 (0.056 ± 0.032)	0.23 (0.47)
25th	0.18 ± 0.12 (0.20 ± 0.11)	0.14 ± 0.070 (0.14 ± 0.067)	0.19 (0.028)	0.19 ± 0.087 (0.16 ± 0.14)	0.11 ± 0.060 (0.13 ± 0.049)	0.11 (0.47)
50th	0.40 ± 0.22 (0.44 ± 0.21)	0.30 ± 0.11 (0.33 ± 0.15)	0.23 (0.027)	0.39 ± 0.17 (0.38 ± 0.27)	0.27 ± 0.14 (0.36 ± 0.14)	0.19 (0.41)
75th	0.90 ± 0.50 (0.80 ± 0.48)	0.64 ± 0.22 (0.60 ± 0.19)	0.13 (0.074)	0.82 ± 0.42 (0.72 ± 0.33)	0.56 ± 0.24 (0.69 ± 0.22)	0.15 (0.47)
90th	1.67 ± 1.05 (1.53 ± 0.89)	1.03 ± 0.32 (1.08 ± 0.28)	0.074 (0.055)	1.48 ± 0.65 (1.10 ± 0.54)	0.81 ± 0.32 (1.42 ± 0.95)	0.023 (0.29)
**Horizontal slow phase velocity (°/s)—subjects with nystagmus**						
10th	0.17 ± 0.21 (0.20 ± 0.15)	0.17 ± 0.13 (0.16 ± 0.13)	0.15 (0.11)	0.14 ± 0.15 (0.088 ± 0.081)	0.15 ± 0.12 (0.17 ± 0.16)	0.50 (0.25)
25th	0.30 ± 0.25 (0.41 ± 0.19)	0.36 ± 0.29 (0.36 ± 0.27)	0.15 (0.23)	0.26 ± 0.22 (0.25 ± 0.13)	0.33 ± 0.22 (0.33 ± 0.27)	0.46 (0.35)
50th	0.55 ± 0.37 (0.74 ± 0.17)	0.62 ± 0.41 (0.66 ± 0.38)	0.15 (0.23)	0.47 ± 0.30 (0.53 ± 0.28)	0.57 ± 0.32 (0.57 ± 0.35)	0.22 (0.50)
75th	0.86 ± 0.43 (1.19 ± 0.29)	0.96 ± 0.56 (0.96 ± 0.44)	0.2891 (0.055)	0.70 ± 0.40 (0.86 ± 0.34)	0.87 ± 0.38 (0.94 ± 0.55)	0.080 (0.42)
90th	1.20 ± 0.50 (1.79 ± 0.60)	1.25 ± 0.67 (1.42 ± 0.58)	0.34 (0.078)	1.01 ± 0.42 (1.44 ± 059)	1.29 ± 0.54 (1.34 ± 060)	0.032 (0.34)

### Fixation stability before and after strabismus repair

We have previously reported that patients with strabismus have greater fixation instability compared to controls. The instability is greater in the NVE compared to the VE during both fellow and amblyopic eye viewing conditions (Table 4). A two-way mixed ANOVA was run to determine the effect of strabismus repair on fixational stability of the VE and NVE as measured using log10 [BCEA (deg^2^)] in patients without and with nystagmus during fellow eye viewing condition. Mauchly's test of sphericity indicated that the assumption of sphericity was met. There was no statistically significant main effect on fixation instability of the VE before and after strabismus repair *F* (1, 16) = 1.91, *p* = 0.18, partial η^2^ = 0.113 with no interaction between the strabismus repair and fixation instability in patients without and with nystagmus, *F* (1, 16) = 1.92, *p* = 0.18, partial η^2^ = 0.114. Similar to VE, there was no statistically significant main effect on fixation instability of the NVE before and after strabismus repair *F* (1, 16) = 0.86, *p* = 0.36, partial η^2^ = 0.05 with no interaction between the strabismus repair and fixation instability in patients without and with nystagmus, *F* (1, 16) = 0.016, *p* = 0.90, partial η^2^ = 0.001.

**Table 4:** Bivariate contour ellipse analysis (log10 [BCEA (deg^2^)]) in viewing eye and non-viewing eye (non-viewing eye in parenthesis) of subjects without and with nystagmus, before and after strabismus repair.

Log BCEA 95% (log10 [BCEA (deg^2^)])			
	No nystagmus	With nystagmus	p value
**Amblyopic eye viewing**			
Preop	0.38 ± 0.24 (0.85 ± 0.27)	0.50 ± 0.69 (1.19 ± 0.55)	0.65 (0.13)
Postop	0.43 ± 0**.**32 (0.76 ± 0.31)	0.25 ± 0.38 (1.04 ± 0.44)	0.33 (0.17)
**Fellow eye viewing**			
Preop	0.39 ± 0.31 (1.02 ± 0.36)	0.26 ± 0.45 (1.11 ± 0.41)	0.51 (0.67)
Postop	0.39 ± 0.28 (0.92 ± 0.29)	0.58 ± 0.44 (1.03 ± 0.44)	0.34 (0.56)

A two-way mixed ANOVA was run to determine the effect of strabismus repair on fixational stability of the VE and NVE as measured using logBCEA in patients without and with nystagmus during amblyopic eye viewing condition. Mauchly's test of sphericity indicated that the assumption of sphericity was met. There was no statistically significant main effect on fixation instability of the VE before and after strabismus repair *F* (1, 16) = 0.51, *p* = 0.48, partial η^2^ = 0.036 with no interaction between the strabismus repair and fixation instability in patients without and with nystagmus, *F* (1, 16) = 1.22, *p* = 0.28, partial η^2^ = 0.08. Similar to VE, there was no statistically significant main effect on fixation instability of the NVE before and after strabismus repair *F* (1, 16) = 0.96, *p* = 0.34, partial η^2^ = 0.06 with no interaction between the strabismus repair and fixation instability in patients without and with nystagmus, *F* (1, 16) = 0.092, *p* = 0.76, partial η^2^ = 0.007.

## Discussion

The main findings of our study are 1) patients with nystagmus were more likely to have poor stereopsis recovery despite improved eye alignment, 2) there was a decrease in the amplitude of the fixational saccades with mild reduction in inter-saccadic drift velocity of the VE and NVE after strabismus repair in patients without nystagmus, 3) patients with nystagmus exhibited no significant change in the fixation instability or median velocity of slow phases after two horizontal muscles surgery. We will broadly divide our discussion into strabismus surgery outcomes and changes in FEMs in patients without and with nystagmus.

The first goal of our study was to report the clinical outcomes in patients without and with nystagmus, namely change in strabismus angle, stereopsis, and monocular visual acuity and number of strabismus surgeries required to achieve good anatomical alignment. Stereopsis development begins at around 6 months of age and continues through early childhood. Studies have shown that early surgery, with reduced periods of duration of misalignment, may preserve stereopsis in children with infantile strabismus^27–32^. Tychsen and colleagues have shown that the severity of latent (fusion maldevelopment) nystagmus is associated with duration of binocular decorrelation in early infancy^11^. Thus, the literature from strabismic non-human primates and human studies to date suggest that the absence of stereopsis after strabismus repair could be due to longer duration of misalignment as well as lack of alignment during early critical periods of visual maturation. The age of onset of esotropia in human studies is determined based on history and chart review. The presence of subtle nystagmus especially during monocular viewing conditions can be difficult to recognize on clinical exam^33^.

Our study is the first to systematically evaluate the effect of strabismus repair on fixational eye movements in human patients, comparing outcomes in those with and without nystagmus. We evaluated the FEM traces to assess for presence of nystagmus, which is a marker of binocular de-correlation in early infancy and assessed the strabismus surgery outcomes. Six of the twelve patients with nystagmus required multiple surgeries—however the age at first strabismus surgery in all the patients was after infancy. In our cohort, we found that patients with nystagmus were less likely to have improvement of stereopsis despite improvement in strabismus angle. We also found that strabismic patients with nystagmus were more likely to have amblyopia (mild: n = 4, moderate: n = 4 and severe: n = 0), whereas none of the patients without nystagmus had amblyopia. In a recent paper from our lab, we have systematically evaluated stereopsis in patients with and without nystagmus as a function of severity of amblyopia. We found that mild to moderate amblyopic patients with nystagmus were more likely to have absent stereopsis compared to patients without nystagmus^34^. We have also previously reported that amblyopic patients with microstrabismus and nystagmus were more likely to have poor recovery of stereopsis and require longer duration of amblyopia treatment despite improvement in visual acuity with patching treatment^19,23,35^. Thus, in agreement with the previous studies from our lab, in the current study we found that patients with nystagmus were more likely to have poor stereopsis recovery after strabismus repair despite having mild to moderate amblyopia. Thus, eye movement recordings can be used as a supplemental tool and provide information on likelihood of regaining stereopsis post-strabismus repair, particularly in older subjects where historic data alone would be less reliable.

Zubcov et al. have shown that strabismus surgery repair can improve the binocular visual acuity^36^. Dell’ Osso et al. have shown that four muscle tenotomy or large recessions result in reduced slow phase velocity of infantile nystagmus with improvement in visual acuity^37^. None of the patients in our cohort had increasing velocity suggestive of infantile nystagmus. We did not systematically measure monocular visual acuity at various gaze angles or binocular visual acuity and binocular fixation instability. With these limitations in mind, in our cohort we did not see any significant change in monocular visual acuity after strabismus repair. The lack of change in visual acuity in our study could be attributed to several factors including monocular viewing conditions, presence of amblyopia, and evaluation of eye movement recordings before and after strabismus repair of only two horizontal muscles, usually of the amblyopic eye.

Behavioral and neurophysiologic studies in humans and non-human primates have shown that saccades and fixational saccades represent an oculomotor continuum^38^, and are generated by common neural circuits^39–42^. NHP studies have shown that the fixational saccades and quick phases of nystagmus are generated within the rostral superior colliculus, whereas the larger visually guided saccades are generated within the caudal superior colliculus^40,43–49^. NHP studies have also provided important insights into the neural correlates of strabismus. Electrical micro-stimulation of the superior colliculus in strabismic NHPs has shown to evoke disconjugate saccades (both in direction and amplitude)^48^. Neurons within the supraoculomotor area, which encodes vergence responses in normal animals, were found to encode horizontal misalignment in strabismic monkeys^12,15,50^. The cells within the rostral superior colliculus have also shown to carry signals related to horizontal eye misalignment and fixation preference in strabismic NHPs^45,46,49^. Human and NHP studies have also shown that strabismus results in increased fixation instability that arises due to the presence of nystagmus and abnormal vergence with resultant alterations of physiologic FEMs^10,12,18,51,52^. We have previously parsed the fast and slow FEMs and have reported FEM abnormalities that correlate with the strabismus angle and extent of binocular function deficits^7^.

Longitudinal studies of strabismus repair in non-human primates have demonstrated small changes in saccades and smooth—pursuit eye movements^53^. Prior human studies have found that the disconjugacy of visually guided saccades decreases to normal values after surgical strabismus repair^17^. In the current paper we examined the changes in FEMs after strabismus repair. We found that in patients without nystagmus there was a mild reduction in the amplitude of the fixational saccades of the VE and NVE as well as reduced inter-saccadic drifts particularly of the NVE after strabismus repair. The majority of patients without nystagmus also had improvement in stereopsis. Strabismus surgery has shown to improve the accuracy of vergence movements and saccades at close distance, and increased the speed of pure convergence and divergence combined movements^54^. The improvement in vergence has shown to correlate with reduced post saccadic drifts^54^. Thus, we speculate that the improvement in FEM abnormalities of viewing and non-viewing eye in patients without nystagmus could be a result of improved vergence. This is also supported by the greater improvement in stereopsis after strabismus repair in patients without nystagmus. We found no significant change in quick phase amplitude or slow phase velocities in patients with nystagmus. In our cohort, we found that few patients with nystagmus had improvement in stereopsis despite improvement in the strabismus angle. Kelly et al. have shown that patients with infantile esotropia were more likely to have congenital impairment of disparity vergence^55^. Also, studies of outcomes of strabismus surgery in infantile esotropia have shown that duration of misalignment is a critical factor influencing the stereopsis outcomes^29,30,32^. Thus, we attribute the lack of improvement in FEM abnormalities after strabismus repair in patients with nystagmus to early onset of disruption of binocularity with diminished/lack of central adaptive mechanisms. Previous studies have found that fixation instability in strabismus, as measured by BCEA, a global measure of dispersion of eye position, is only partially explained by increased amplitude and more frequent fixational saccades^52^. We did not find any difference in BCEA values before and after strabismus repair in patients with and without nystagmus. This is in agreement with a strabismic NHP study where no consistent changes of fixation instability were reported post strabismus repair^53,56^.

In conclusion, we examined the clinical outcomes following strabismus repair as a function of fixation eye movement waveforms, fast and slow eye movement parameters, and fixation instability. The analysis allowed us to examine the treatment response to strabismus repair in patients with and without nystagmus. The results highlight the importance of systematically evaluating the FEM traces for accurate diagnosis of presence of nystagmus and analyzing the slow and fast eye movement characteristics. The data from the current study suggest that eye movement characterization and quantification can serve as an important tool to assess functional outcomes after strabismus repair. Future neurophysiologic and behavioral studies incorporating fixation eye movement assessments will provide further insights into the neural versus peripheral mechanisms driving the strabismus, neural plasticity, and adaptation following strabismus surgery.

## References

[1] Barlow HB (1952). Eye movements during fixation. J. Physiol..

[2] Ditchburn RW, Ginsborg BL (1953). Involuntary eye movements during fixation. J. Physiol..

[3] Ginsborg BL, Maurice DM (1959). Involuntary movements of the eye during fixation and blinking. Br. J. Ophthalmol..

[4] Bucci MP, Kapoula Z, Eggert T, Garraud L (1997). Deficiency of adaptive control of the binocular coordination of saccades in strabismus. Vis. Res..

[5] Kapoula Z, Bucci MP, Eggert T, Garraud L (1997). Impairment of the binocular coordination of saccades in strabismus. Vis. Res..

[6] Ghasia FF, Shaikh AG, Jacobs J, Walker MF (2015). Cross-coupled eye movement supports neural origin of pattern strabismus. Invest. Ophthalmol. Vis. Sci..

[7] Ghasia FF, Otero-Millan J, Shaikh AG (2018). Abnormal fixational eye movements in strabismus. Br. J. Ophthalmol..

[8] Tychsen L (2007). Causing and curing infantile esotropia in primates: The role of decorrelated binocular input (an American Ophthalmological Society thesis). Trans. Am. Ophthalmol. Soc..

[9] Tychsen L, Richards M, Wong A, Foeller P, Bradley D, Burkhalter A (2010). The neural mechanism for Latent (fusion maldevelopment) nystagmus. J. Neuroophthalmol..

[10] Tychsen L, Scott C (2003). Maldevelopment of convergence eye movements in macaque monkeys with small- and large-angle infantile esotropia. Invest. Ophthalmol. Vis. Sci..

[11] Richards M, Wong A, Foeller P, Bradley D, Tychsen L (2008). Duration of binocular decorrelation predicts the severity of latent (fusion maldevelopment) nystagmus in strabismic macaque monkeys. Invest. Ophthalmol. Vis. Sci..

[12] Das VE (2012). Responses of cells in the midbrain near-response area in monkeys with strabismus. Invest. Ophthalmol. Vis. Sci..

[13] Economides JR, Rapone BC, Adams DL, Horton JC (2018). Normal topography and binocularity of the superior colliculus in strabismus. J. Neurosci..

[14] Das VE (2016). Strabismus and the oculomotor system: Insights from Macaque models. Annu. Rev. Vis. Sci..

[15] Walton MMG, Pallus A, Fleuriet J, Mustari MJ, Tarczy-Hornoch K (2017). Neural mechanisms of oculomotor abnormalities in the infantile strabismus syndrome. J. Neurophysiol..

[16] Bucci MP, Vernet M, Gerard CL, Kapoula Z (2009). Normal speed and accuracy of saccade and vergence eye movements in dyslexic reader children. J. Ophthalmol..

[17] Bucci MP, Kapoula Z, Yang Q, Roussat B, Bremond-Gignac D (2002). Binocular coordination of saccades in children with strabismus before and after surgery. Invest. Ophthalmol. Vis. Sci..

[18] Kang SL, Beylergil SB, Otero-Millan J, Shaikh A, Ghasia F (2019). Fixational eye movement waveforms in amblyopia: Characteristics of fast and slow eye movements. J. Eye Mov. Res..

[19] Scaramuzzi M, Murray J, Otero-Millan J, Nucci P, Shaikh AG, Ghasia FF (2020). Part time patching treatment outcomes in children with amblyopia with and without fusion maldevelopment nystagmus: An eye movement study. PLoS ONE.

[20] Shaikh AG, Otero-Millan J, Kumar P, Ghasia FF (2016). Abnormal fixational eye movements in amblyopia. PLoS ONE.

[21] McCamy MB, Otero-Millan J, Macknik SL (2012). Microsaccadic efficacy and contribution to foveal and peripheral vision. J. Neurosci..

[22] Troncoso XG, Macknik SL, Martinez-Conde S (2008). Microsaccades counteract perceptual filling-in. J. Vis..

[23] Scaramuzzi M, Murray J, Nucci P, Shaikh AG, Ghasia FF (2021). Fixational eye movements abnormalities and rate of visual acuity and stereoacuity improvement with part time patching. Sci. Rep..

[24] Steinman RM, Cushman WB, Martins AJ (1982). The precision of gaze. A review. Hum. Neurobiol..

[25] Martinez-Conde S (2006). Fixational eye movements in normal and pathological vision. Prog. Brain Res..

[26] Shaikh AG, Ghasia FF (2017). Fixational saccades are more disconjugate in adults than in children. PLoS ONE.

[27] Birch E, Stager D, Wright K, Beck R (1998). The natural history of infantile esotropia during the first six months of life. Pediatric Eye Disease Investigator Group. J. AAPOS.

[28] Birch EE, Fawcett S, Stager DR (2000). Why does early surgical alignment improve stereoacuity outcomes in infantile esotropia?. J. AAPOS.

[29] Birch EE, Stager DR, Berry P, Everett ME (1990). Prospective assessment of acuity and stereopsis in amblyopic infantile esotropes following early surgery. Invest. Ophthalmol. Vis. Sci..

[30] Ing MR, Okino LM (2002). Outcome study of stereopsis in relation to duration of misalignment in congenital esotropia. J. AAPOS.

[31] Ing MR (1991). Early surgery for essential infantile esotropia. J. Pediatr. Ophthalmol. Strabismus.

[32] Wright KW, Edelman PM, McVey JH, Terry AP, Lin M (1994). High-grade stereo acuity after early surgery for congenital esotropia. Arch. Ophthalmol..

[33] Abadi RV, Dickinson CM (1986). Waveform characteristics in congenital nystagmus. Doc. Ophthalmol..

[34] Murray J, Garg K, Ghasia F (2021). Monocular and binocular visual function deficits in amblyopic patients with and without fusion maldevelopment nystagmus. Eye Brain.

[35] Scaramuzzi M, Murray J, Otero-Millan J, Nucci P, Shaikh AG, Ghasia FF (2019). Fixation instability in amblyopia: Oculomotor disease biomarkers predictive of treatment effectiveness. Prog. Brain Res..

[36] Zubcov AA, Stark N, Weber A, Wizov SS, Reinecke RD (1993). Improvement of visual acuity after surgery for nystagmus. Ophthalmology.

[37] Dell’Osso LF, Flynn JT (1979). Congenital nystagmus surgery A quantitative evaluation of the effects. Arch. Ophthalmol..

[38] Otero-Millan J, Macknik SL, Langston RE, Martinez-Conde S (2013). An oculomotor continuum from exploration to fixation. Proc. Natl. Acad. Sci. USA.

[39] Otero-Millan J, Macknik SL, Serra A, Leigh RJ, Martinez-Conde S (2011). Triggering mechanisms in microsaccade and saccade generation: A novel proposal. Ann. N. Y. Acad. Sci..

[40] Hafed ZM, Goffart L, Krauzlis RJ (2009). A neural mechanism for microsaccade generation in the primate superior colliculus. Science.

[41] Rolfs M, Kliegl R, Engbert R (2008). Toward a model of microsaccade generation: The case of microsaccadic inhibition. J. Vis..

[42] Steinman RM, Haddad GM, Skavenski AA, Wyman D (1973). Miniature eye movement. Science.

[43] Schiller PH, Stryker M (1972). Single-unit recording and stimulation in superior colliculus of the alert rhesus monkey. J. Neurophysiol..

[44] Robinson DA (1972). Eye movements evoked by collicular stimulation in the alert monkey. Vis. Res..

[45] Upadhyaya S, Das VE (2019). Response properties of cells within the rostral superior colliculus of strabismic monkeys. Invest. Ophthalmol. Vis. Sci..

[46] Upadhyaya S, Meng H, Das VE (2017). Electrical stimulation of superior colliculus affects strabismus angle in monkey models for strabismus. J. Neurophysiol..

[47] Economides JR, Adams DL, Horton JC (2016). Normal correspondence of tectal maps for saccadic eye movements in strabismus. J. Neurophysiol..

[48] Fleuriet J, Walton MM, Ono S, Mustari MJ (2016). Electrical microstimulation of the superior colliculus in strabismic monkeys. Invest. Ophthalmol. Vis. Sci..

[49] Van Horn MR, Waitzman DM, Cullen KE (2013). Vergence neurons identified in the rostral superior colliculus code smooth eye movements in 3D space. J. Neurosci..

[50] Das VE (2011). Cells in the supraoculomotor area in monkeys with strabismus show activity related to the strabismus angle. Ann. N. Y. Acad. Sci..

[51] St Cyr GJ, Fender DH (1969). The interplay of drifts and flicks in binocular fixation. Vision. Res..

[52] Upadhyaya S, Pullela M, Ramachandran S, Adade S, Joshi AC, Das VE (2017). Fixational saccades and their relation to fixation instability in strabismic monkeys. Invest. Ophthalmol. Vis. Sci..

[53] Pullela M, Degler BA, Coats DK, Das VE (2016). Longitudinal evaluation of eye misalignment and eye movements following surgical correction of strabismus in monkeys. Invest. Ophthalmol. Vis. Sci..

[54] Bucci MP, Bremond-Gignac D, Kapoula Z (2009). Speed and accuracy of saccades, vergence and combined eye movements in subjects with strabismus before and after eye surgery. Vis. Res..

[55] Kelly KR, Felius J, Ramachandran S, John BA, Jost RM, Birch EE (2016). Congenitally impaired disparity vergence in children with infantile esotropia. Invest. Ophthalmol. Vis. Sci..

[56] Pullela M, Agaoglu MN, Joshi AC, Agaoglu S, Coats DK, Das VE (2018). Neural plasticity following surgical correction of strabismus in monkeys. Invest. Ophthalmol. Vis. Sci..

